# Barriers and Facilitators to Screening for Cognitive Impairment in Australian Rural Health Services: A Pilot Study

**DOI:** 10.3390/geriatrics7020035

**Published:** 2022-03-22

**Authors:** Sean MacDermott, Rebecca McKechnie, Dina LoGiudice, Debra Morgan, Irene Blackberry

**Affiliations:** 1 John Richards Centre for Rural Ageing Research, La Trobe Rural Health School, La Trobe University, Wodonga, VIC 3690, Australia; r.mckechnie@latrobe.edu.au (R.M.); i.blackberry@latrobe.edu.au (I.B.); 2 Department of Aged Care, Royal Melbourne Hospital, Melbourne, VIC 3050, Australia; dina.logiudice@mh.org.au; 3 Canadian Centre for Health and Safety in Agriculture (CCHSA), University of Saskatchewan, Saskatoon, SK S7N 2Z4, Canada

**Keywords:** cognitive impairment, cognitive screening, older people, rural health

## Abstract

Australian National standards recommend routine screening for all adults over 65 years by health organisations that provide care for patients with cognitive impairment. Despite this, screening rates are low and, when implemented, screening is often not done well. This qualitative pilot study investigates barriers and facilitators to cognitive screening for older people in rural and regional Victoria, Australia. Focus groups and interviews were undertaken with staff across two health services. Data were analysed via thematic analysis and contextualized within the i-PARIHS framework. Key facilitators of screening included legislation, staff buy-in, clinical experience, appropriate training, and interorganisational relationships. Collaborative implementation processes, time, and workloads were considerations in a recently accredited tertiary care setting. Lack of specialist services, familiarity with patients, and infrastructural issues may be barriers exacerbated in rural settings. In lieu of rural specialist services, interorganisational relationships should be leveraged to facilitate referring ‘outwards’ rather than ‘upwards’.

## 1. Introduction

Australia’s older adult population will more than double to 8.8 million (22%) by 2057, increasing from 3.8 million in 2017. Corresponding with an increase in aging will be an increase in age-related comorbidities and resultant burden on the healthcare system, and the impact will be disproportionately experienced in rural areas, which boast a higher proportion of older adults (34%) compared to urban locations (28%). This is of particular concern given already poorer availability and distribution of healthcare services and staff in rural and regional areas [1]. 

One such age-related health consideration is cognitive impairment (CI), which has significant implications for the healthcare requirements of older people. As people age, structural and functional changes of the brain can lead to CI [2]. CI refers to changes in cognition across a spectrum ranging from normal age-related cognitive decline through to severe CI and includes, but is not limited to, diagnoses such as dementia and delirium [3,4]. Mild Cognitive Impairment (MCI) is considered an intermediate state between normal cognitive decline with aging and dementia [5]. In complex acute-care settings, proper diagnosis of dementia may be challenging due to the presence of other causes of memory loss and thinking disorders such as delirium, head injury or narcosis, which are symptomatically similar to dementia [6], and full diagnostics for assessment may not be feasible or appropriate in all settings. Instead, the term ‘cognitive impairment’ (CI) is adopted to describe issues of memory and thinking seen in patients without the requirement to ascribe a diagnosis, and simple cognition screening tests can be used for detection [7]. 

CI is a relatively common occurrence in older adults (65 years and older), particularly during hospitalisation [8]. There is little evidence relating to actual rates of cognitive deficit among older people in general, as most statistics focus on dementia as the more severe form. Previous studies suggest that approximately 3.7% of older adults experience MCI, and approximately 2% are diagnosed with dementia [9,10]. These percentages increase for older people who are hospitalised, for which 40% will screen positive for CI [11,12]. In light of these statistics, the focus of screening is directed at tertiary hospital settings, but it is also important for the broader health workforce to be able to identify CI in order to seek avenues of treatment and support for older people [13]. 

CI is frequently under-recognised and under-documented [6]. Failure to effectively screen for and identify CI may result in inappropriate or insufficient care. Patients with CI have higher support needs for communication, ambulation, toileting and medication administration, but may not have the assumed cognitive ability to comply with care directives (for example medication regimes) and are also less likely to be able to communicate their needs [14,15]. This contributes to poorer health outcomes, including increased risk of falls and poor functional outcomes, psychological distress in unfamiliar environments, higher risk of hospital-acquired complications, increased length of stay and readmission (LOS) and higher mortality [7,16,17,18,19,20]. Identifying CI is therefore a key strategy for improving the care of older adults and reducing financial burden on the healthcare system, with previous research suggesting that effective screening and identification contributed to a reduction in costs of nearly $400 per patient screening positive [11]. 

The Australian Commission on Safety and Quality in Healthcare has stipulated actions in the National Safety and Quality Health Service (NSQHS) Standards and has released the Delirium Clinical Care Standard [13,21,22]. These standards dictate that patients aged over 65, or over 50 years for Aboriginal and Torres Strait Islander people, are an at-risk population for whom routine cognitive screening should be embedded into practice [13,23]. Despite this, screening rates in hospital settings are typically less than 60% and may be lower in other community health settings. Where screening does occur it is often not done well or consistently [11]. Complexities around identifying CI may explain poor adoption rates and/or use of screening. Defining subclinical CI can be challenging due to lack of diagnostic criteria and disagreement regarding scale cut-off points to determine cognitive impairment [24]. Thus, those at the mild end of the spectrum of cognitive deficit may not be diagnosed during routine care. This may be exacerbated by the lack of a standard set of measures and differential characterisation of MCI based on the type of tool used, compounded by differences in training and exposure of staff in using available tools [11,25]. 

There is a notable paucity of research investigating barriers to cognitive screening for older people, particularly in rural settings [26], and to our knowledge no studies have focused on rural Australian populations. In other health settings, onerous tools, time constraints associated with documentation and communication, limited human resources, a lack of specialists for referral and treatment post-diagnosis, and reluctance by practitioners and patients to discuss uncomfortable issues have previously been identified as potential barriers to screening for cognitive impairment [16,26,27]. 

In light of projected increases to the older Australian population and the concomitantly enhanced burden on healthcare systems, it is imperative that we identify the facilitators and barriers to CI screening. It should not be assumed that the barriers to cognitive screening as identified in other contexts also apply in rural areas. Given the higher proportion of older adults in rural locations it is essential to identify factors that that are specific, or more applicable, to screening of older populations in those areas. The aim of this pilot study is, therefore, to identify and develop a preliminary understanding of barriers and enablers to the implementation and maintenance of Cognitive Impairment (CI) screening of patients aged over 65 in rural or regional Health Services to develop recommendations for more effective facilitation of screening programs.

## 2. Materials and Methods

### 2.1. Evaluation Framework

This analysis was guided by the i-PARIHS Framework [28], a conceptual framework to both explain and predict why the implementation of evidence into practice is, or is not, successful through its consideration of aspects across three core constructs;

- Innovation, considers the characteristics of knowledge and how these affect its practical application;
- Recipients, considers the impact of individuals or groups of individuals in supporting or resisting innovation;
- Inner and outer contexts, considers the resources, culture, leadership, policy and capacity for innovation at both the local and wider organisational levels.

The i-PARIHS framework contextualises these constructs with facilitation as the active element that assesses, aligns, and integrates them to produce successful implementation or uptake of innovation in practice (Successful Implementation = Facilitation (Implementation + Recipients + Context) [28]. The characteristics of each construct to be considered within the i-PARIHS framework are summarised in Table 1.

### 2.2. Study Design and Setting

This paper reports on the qualitative thematic analysis of two focus groups and three interviews with health professionals across two health services in a regional Victorian town in Australia. 

#### Participant Recruitment and Data Collection

Participating organisations were the main community/primary healthcare service and one of two main tertiary healthcare providers servicing a sparsely populated Local Government Area (LGA) of 55,515 people and incorporating regions classified as either ‘Outer Regional’ or ‘Remote’ by the Australian Statistical Geography Standard—Remoteness Areas typology [29]. A second tertiary healthcare provider had initially agreed to participate but withdrew prior to data collection due to significant organisational change. We contacted each healthcare provider to request that study details be sent to staff who had expertise in or were responsible for administering cognitive screening, or were involved with accreditation of screening standards.

A key contact in each service was alerted to the project via an e-mail requesting expressions of interest in participating. They in turn disseminated the expression of interest to the aforementioned staff members involved in cognitive screening and/or accreditation standards. Those interested in participating contacted the CI directly via e-mail. Two focus groups were scheduled (one per service), and three interviews for participants unable to attend the focus groups. Participation comprised a single 1.5-h focus group or interview. A semi-structured interview schedule was employed with questions related to understanding of NSQHS and requirements around CI, current screening practices, and barriers and facilitators. Focus Groups were co-facilitated by the Chief Investigator (first author) and a research assistant, who also conducted the supplementary interviews using the same question schedule. A copy of the interview schedule is provided as Supplementary Materials. A summary of focus group and interview attendants is included in Table 2. Twenty-two people participated (7 tertiary setting, 15 primary/community care), spanning disciplines of gerontology, nursing, and allied health.

### 2.3. Data Analysis

Interviews were audio-recorded and transcribed verbatim. Data were analysed as per the phases outlined by Braun and Clarke: (1) Reviewers familiarised themselves with the data, (2) transcripts were broken down into the smallest meaningful segments of text and initial codes were inductively assigned, (3) initial codes were grouped to develop themes, (4) potential themes were reviewed, (5) themes were defined [30]. Details regarding the occupation or position of individual sources of each quote were not included in the results section due to the potential for identification of participants from these small regional healthcare teams.

A deductive approach was then adopted whereby the themes and sub-themes were reviewed in order to assign each into one or more domains of the i-PARIHS framework and to contextualise these within the three overarching constructs. Rigour of the findings was supported by two different researchers independently undertaking the above process, followed by comparison and discussion of the respective findings, during which coding and the ascribed i-PARIHS domains were compared and categories continuously refined through an iterative process. 

## 3. Results

The overarching themes identified and the sub-themes underpinning them are summarised in Table 3 and grouped as barriers or enablers at the end of this section in Table 4.

### 3.1. Themes Identified

#### 3.1.1. Legislation

Legislation was identified as a key facilitator for adoption. Mandates or support by official or governing bodies, such as the recent NSQHS, was a key reason for the adoption of cognitive screening in the tertiary setting. This also necessitated the development of organizational policy to guide the application of screening protocols. Conversely, in the absence of mandated screening, the community health service was not routinly undertaking cognitive screening.

*‘It [screening] is now a requirement that we’re mandated to do it.’… FG1.*

#### 3.1.2. ‘Staff Buy-In’

The acceptance of screening programs appeared to be based on staff buy-in, underpinned by several sub-themes: ‘nothing new’, ‘collaborative journey’, ‘meaning and purpose’, a ‘systematic approach to enhance practice and make it easier’, and ‘time and workloads as barriers to practice’.

Meaning and purpose was a strong sub-theme across both organisations; however, it was experienced on different ends of the spectrum in the tertiary care facility compared to the community setting. Within the tertiary care setting, staff shared a clear understanding of why screening protocols were in place and a shared purpose for screening that generated support and appreciation for the process. The establishment of this shared understanding in relation to meaning and purpose appeared in part to be underpinned by the collaborative journey through which formal screening policy and practices were introduced. This process involved gradual, ongoing consultation to develop the screening protocol and training to facilitate a consistent purpose and understanding. Contributions and experiences by all staff were welcomed and valued, and as such staff were empowered and had ownership over the screening process. Conversely, for staff based in primary or community care settings, meaning or purpose of screening appeared less clear or established, perhaps due to lack of opportunity to address the results with subsequent referrals or follow-up (discussed further below under ‘ancillary services and support’).

*‘It’s been like a group involvement… we come to the meetings …we’ve sort of all been gradually introduced to it.’… positive point where we think that it’s right.’… FG1.*

*‘The education was really more about bringing people on the journey, not just introducing another piece of paper, which would have just—we would have had a mutiny.’… ‘That’s what I encourage… is that this is not just another bit of paper.’… FG1.*

*‘The other thing is that so what factor, so what can we do afterwards? Like if the results show a cognitive impact… there’s a lack of services to actually put something into place [for that] person.’… FG2.*

The adoption of formal screening protocols within the tertiary setting seemed to be more acceptable due to perceptions of it being nothing new. Staff had already been routinely screening patients, and therefore the new program leveraged existing practice as well as providing explicit instructions or tools to support it.

*‘It’s not something additional that you’re doing because you’re already doing that.’… FG1.*

Formalising existing practice also provided a systematic process towards screening, which was perceived to make existing tasks easier, create efficiencies and eliminate errors associated with guessing or assuming client diagnoses and requirements. Ultimately, this was perceived to streamline workflows and continuity of care for patients. The importance of leveraging existing practice and a systematic approach as enablers to screening were reinforced by the identification of time and workloads as potential barriers in the tertiary care setting.

*‘To have something that you can actually tick and say, yes, I’ve done this, yes, I’ve covered that, I’ve excluded this, I’ve included—thought about that.’…… FG1.*

*‘There isn’t often a lot of time and we need to get that value out of whatever tool we’re going to use’.… ‘It’s a never-ending paper trail again.’… FG1.*

#### 3.1.3. Ancillary Services and Support

The availability of support services was an important consideration in the next step of the process (i.e., in addressing the results of screening) and thus had the potential to be both a barrier and enabler to screening. In this regional setting a lack of specialist services to refer to when identifying CI proved a barrier to adopting screening; practitioners did not see the benefits of undertaking screening when opportunities to provide support for people who screened positive for CI were limited or non-existent. However, the closeness and familiarity offered in smaller regional settings also promoted close inter-organisational relationships with other community support services, thereby providing potential to circumvent the effects of a scarcity of specialists, filling this gap to provide support and capacity to assist clients experiencing CI and thus facilitating the adoption of cognitive screening protocols.

*‘If we do identify that deficit what do we do with it and who do—we don’t have access to a neuropsych. Trying to get access to an OT or psychologist is very difficult.’… I1.*

*‘We have access to a lot of good support systems… to the supports we need for people, and we also have very good relationships with. So in some ways I think being smaller is easier for everyone to know what’s happening.’… I2.*

#### 3.1.4. Tool Availability

Ready availability of validated tools relevant to older people contributed to perceived ease and acceptability of cognitive screening. Practitioners referred to the evidence base (i.e., available literature) to identify valid and reliable tools, with consideration given to their appropriateness for the specific context and target group in which they were to be applied. Concerns regarding tool validity were evident, with some perceived as unnecessarily difficult or irrelevant for older people, and potentially not ‘tapping in’ to the correct constructs to indicate CI.

*‘The Australian hospital guides and in that—the standards, it gives you a list of tools that are the best practice tools.’… FG1.*

*‘One of the tests, to be honest, we’re all worried about failing ourselves.’… FG1.*

*‘There can also be concern that clients may memorise the test, like for example the mini-mental state examination that’s run often in hospitals.’… FG2.*

#### 3.1.5. Reliance on Clinical Experience

Reliance on clinical experience presented as both a potential barrier and facilitator to screening. On one end of the spectrum, participants from primary and community services relied on clinical experience to identify when a participant should be screened, in place of screening all participants based on pre-defined criteria. In this way clinical experience was an important aspect which enhanced screening processes, with many participants reflecting on changes to eligibility criteria based on understanding of and experience with their clientele and how cognitive function was affected amongst them. This allowed practitioners to apply a more holistic lens whereby considerations of eligibility extended beyond just the age range specified.

*‘Screening is generally in-home and observation of people functioning in their own environment. Then if it flagged … they would go on and use a more formalised [tool].’… I3.*

*‘I’ll just make a point that in our cohort of people with mental illness and drug and alcohol problems, 65 is probably a little bit too old. I’d be looking at over 55 for when you’re wanting to screen for those sorts of things.’… I1.*

Whilst this highlights the importance of clinical experience in cognitive screening, it also illustrates a potential risk associated with overreliance on clinical experience: using subjective observation to determine whether CI screening should occur. This is a particular risk for people with MCI who are more likely to present as functioning normally: a risk that may be exacerbated in smaller rural settings where clinicians are familiar with clients.

#### 3.1.6. Training and Proficiency

Training and proficiency in screening were identified as important facilitators to screening in both tertiary and community contexts. The benefits of training were encompassed within the sub-theme ‘training = experience = knowledge and confidence’. Training in screening processes and methodologies enhanced knowledge understanding and perceived purpose of screening. There were also purported benefits for confidence that improved practitioner willingness to adopt screening practices.

*‘And we did them [screening] on each other and that was really helpful, actually having that practice of saying the script.’… ‘Doctors came back and said just do a mini-mental, and I’m like no, I don’t want to do a mini-mental for these reasons. I had the confidence to be able to explain.’… FG2.*

As such, a lack of available training or experience with screening may serve as a barrier to screening practice. In particular, a paucity of screening-related training or experiences in university programs was noted, and most practitioners had been required to seek training opportunities post-graduation and of their own volition.

*‘My degree specifically I probably had two to three weeks max on the cognitive screening.’… FG2.*

#### 3.1.7. Patient Experience as a Motivator 

The patient experience was simultaneously a barrier and a facilitator to screening adoption. In the tertiary setting in which systematic screening processes had already been implemented, staff had directly observed improved patient outcomes, including better continuity of care, and the prevention of premature discharge. Observation of tangible benefits therefore provided justification and motivation to continue screening.

*‘They found the patient didn’t have delirium but it was actually a cognitive impairment that they’d had previous to coming in.’…‘There’s been a mentality of, quick, let’s get them back to their own environment because that’s—they’re going to quickly improve in their own environment when it potentially might not be the case as well.’… FG1.*

Conversely, patient discomfort and concerns regarding negative repercussions from a positive screening result, such as institutionalisation, loss of driving licence and independence (particularly in rural areas where public transportation options are limited) potentially deterred practitioners from screening, in favour of building rapport over following best practice.

*‘A hesitancy to ask them the question if you do know them and they answer incorrectly … It could be sort of embarrassing for them.’… FG1.*

*‘Just a lot of clients are really worried that you’re going to take off their driver’s licence…’… FG2.*

#### 3.1.8. Familiarity

Within small rural tertiary care settings, familiarity with clients may result in practitioners anticipating a specific outcome from screening or failing to identify the need to screen clients based on subjectively observing people functioning within their usual environments. Again, this risk may be particularly exacerbated for those with MCI, who may be more likely to present as functioning ‘normally’, particularly in settings in which they are comfortable and familiar. Additionally, an existing relationship within the smaller communities and social settings offered in smaller regional locations may contribute to the aforementioned hesitancy to screen to avoid embarrassment for the client.

*‘With the smaller hospital, maybe something that could come up is that we know a lot of our patients… we assume that they’re going to be negative or positive for the cognitive impairment.’… FG1.*

### 3.2. i-PARIHS Framework for Successful Implementation

**Figure 1:**
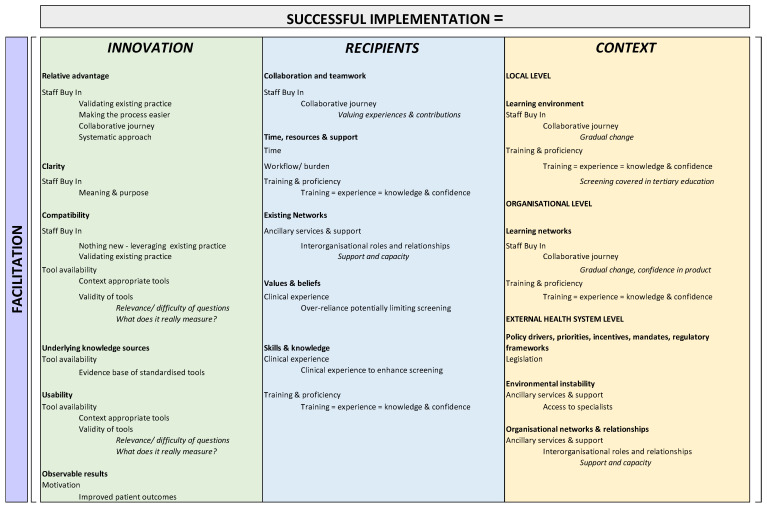
Themes and sub-themes contextualised within i-PARIHS domains.

Following identification of themes, findings were contextualised within the i-PARIHS framework, as highlighted in Table 2 and summarised in Figure 1. Figure 1 depicts the three core constructs that characterise i-PARIHS (innovation, recipients, and context) as individual columns, with the respective underpinning domains as bold headings within each relevant construct.

Overall, the application of the i-PARIHS framework indicates that Successful Implementation (SI) = Facilitation [Legislation + Staff buy-in + Ancillary support services + Training and proficiency + Clinical experience + Tool availability + Training + Observable benefits]. By then, construing our findings within the i-PARIHS framework in Table 1, we can identify areas of focus or points of leverage for successful implementation, and these include:

- Within the **innovation** construct—relative advantage of building on existing process and making current practice easier; clarity from shared purpose and understanding; compatibility with/of existing practices and available tools; existing evidence bases as underlying knowledge sources; careful consideration of existing tools and relevance to the context to enhance usability; and motivation through identifying observable patient benefits.
- Within the **recipient** construct—a collaborative approach in which all experiences and contributions are valued to achieve staff buy-in and therefore collaboration and teamwork; providing sufficient time as well as resources and support through training opportunities; looking to existing networks for support and capacity in light of limited specialist availability; addressing skills and knowledge through training; and leveraging clinical experience to give context to screening protocols.
- Within the **context** construct—creating a supportive learning environment (local and organizational level) and fostering training and sufficiency through gradual change and the provision of training (in the workforce and tertiary education); advocating for legislation to address policy drivers, mandates and regulatory frameworks as drivers of screening adoption; and leveraging organisational supports and relationships to overcome specialist shortages as barriers to addressing the results of screening.

## 4. Discussion

This research sought to identify the facilitators and barriers to cognitive screening for older people in rural care settings. We identified eight key themes underpinning the implementation and adoption of cognitive screening: legislation, staff buy-in, ancillary services and support, tool availability, reliance on clinical experience, training and proficiency, patient experience as a motivator, and familiarity with clients. These themes were in turn underpinned by a number of sub-themes. Whilst many of the themes and sub-themes identified in the current study are similar to those experienced in metropolitan settings, several are potentially unique to, or exacerbated by, a rural setting. We discuss our findings further below, within the context of their saliency within a regional and rural locations. 

### 4.1. Factors Consistent with Previous Studies in Rural and/or Urban Settings

Legislation or policy is a key facilitator for adoption, with NSQHS referred to by multiple participants as the main driver for implementing cognitive screening. In the tertiary setting, established organisational policy relating to the administration of screening and communication of results further supported screening programs. Conversely, the community health setting did not consistently implement screening and appeared to lack policy relating to it. These findings are consistent with previous research, which identified a requirement to screen all over 65, as a significant organisational driver [11]. Since January 2019, Australian Health Services are assessed against NSQHS Standards that identify patients aged over 65 as an at-risk population requiring cognitive screening [13]. Such legislation and accompanying organisational policy may formalise the requirement for screening and make it the ‘status quo’.

At the time that this study was conducted, the tertiary care setting had adopted a formal screening policy. This was facilitated by staff buy-in attained through the development of meaning and purpose associated with screening and was achieved through: (i) collaborative approach to gradual change to empower staff to own the screening process, (ii) fitting in with existing practices to leverage work already being undertaken to (iii) make it easier and validate existing practice. Conversely, the community health setting was not implementing routine screening, which was partly explained by a perceived lack of purpose as a barrier. Our findings align with the broader literature in which provider buy-in and medical staff endorsement facilitated screening [11,31], and staff buy-in and implementation of new practices may be enhanced through training in change processes to enhance organisational readiness for change [32]. 

Time and workloads were constraints to screening in the tertiary but not the community care setting. This is not consistent with the findings of previous research [33] and may have arisen because the primary and community care services in the current research received numerous referrals through a government aged care initiative (My Aged Care), through which cognitive screening was an inherent part of eligibility assessment, thus negating the need for further screening.

### 4.2. Factors Exacerbated in the Current Rural Setting

Training relating to cognitive assessment improved knowledge and confidence in the adoption of screening, and the selection and use of tools. Training and exposure is essential in improving confidence and facilitating implementation of screening programs, as practitioners are reluctant to administer tools with which they are unfamiliar [11,33,34]. Current training programs for healthcare providers incorporate little exposure to screening, resulting in limited confidence to adopt and implement screening practices. Practitioners need more access to training opportunities, particularly those from rural locations in which geographic and financial barriers to training are significant [27,35]. 

An interesting finding of the current research is the double-edged relationship between clinical experience and CI screening. Overreliance on clinical experience may limit the use of screening if practitioners rely on subjective judgement to determine whether cognitive screening should occur. This increases the risk of mild cognitive deficits going unnoticed. This may be particularly exacerbated in rural areas, where healthcare staff have existing relationships with some clients, reflecting the potential for a type of expectation bias to adversely influence the identification of signals for screening. Expectation bias occurs when a person’s expectations about a particular outcome influence perceptions of the individuals’ or others’ behaviours, and in the case of health assessments may alter one’s ability to observe cues that may indicate the need for screening [36,37,38,39]. Conversely, clinical experience has the potential to expand screening practices beyond standard eligibility criteria, due to better understanding of the target group, and how and when cognitive function could be affected. In the current sample this resulted in screening implemented for some patients who fell below the age cut-off of 65 years. 

Reluctance to screen for CI was exacerbated by perceived discomfort (for both patient and practitioner) and the potential for adverse implications associated with a positive result. Patients fear the results of cognitive screening due to perceived negative outcomes such as institutionalisation, and loss of driving license and independence. Medico–legal concerns, for example, fitness to drive and loss of independence, have previously been identified as barriers to screening processes [16], and patients are likely to be particularly uncomfortable discussing issues of cognitive performance due to the social stigma associated with mental health [27]. These issues are likely particularly exacerbated in rural contexts with limited public transport and physical infrastructure, or where values of resiliency and autonomy are core amongst residents [26]. Our findings may point to a misunderstanding of the purpose of cognitive screening, and the need for more clarity about the process, outcomes, and benefits among healthcare staff and clients [26,40].

### 4.3. Factors Salient in the Current Rural Setting

A lack of specialist services was a significant barrier to the adoption of screening programs, due to concomitant lack of opportunity to address positive results. The scarcity of healthcare workers, especially specialists, in rural and regional Australia is well known, and consistent with previous research, uncertainty and lack of a ‘next step’ limit the opportunity to address positive screen results, thus diminishing screening’s perceived purpose and limiting motivation for adoption [26,27]. Conversely, close relationships between support organisations in rural towns partially ameliorated this barrier. This aligns with other findings in which intra- and inter-organisation communication and collaboration have been identified as important to the facilitation of screening program installation and functioning [31]. Such relationships should be fostered and leveraged to provide rural hospitals with opportunities to extend referrals ‘out’ rather than ‘up’ to provide benefits to patients with positive screen results.

A limitation of this research is the participation of just two of the three local health organisations. Future research should reinforce or build upon the current preliminary findings by extending into a more diverse sample of healthcare providers and services in other regional areas. 

## 5. Conclusions

This pilot study identified numerous facilitators and barriers to the adoption of cognitive screening programs. Legislation, staff buy-in (achieved through a collaborative development process and opportunities to leverage and improve existing practice), interorganizational relationship and support, tool availability, clinical experience, training and proficiency, and positive patient experience as motivators were key facilitators of screening. Time and workloads were also important considerations in the tertiary setting. Exacerbated by rural locality, concerns regarding screening results, which were underpinned by a lack of specialist services to refer to, familiarity with patients and the risk of discomfort, and insufficient infrastructure were potential barriers to adopting screening programs. Thus, fostering interorganisational relationships with other community organisations for outwards referrals and providing better access to training may be particularly successful in enhancing the adoption and implementation of cognitive screening programs in rural settings.

## Figures and Tables

**Table 1:** Characteristics of i-PARIHS constructs.

Innovation	Recipients	Context
Underlying knowledge sources Clarity Degree of fit with existing practice and values (compatibility or contestability) Usability Relative advantage Trialability Observable results	Motivation Values and beliefs Goals Skills and knowledge Time, resources, support Local opinion leaders Collaboration and teamwork Existing networks Power and authority Presence of boundaries	Local level: Formal and informal leadership support Culture Past experience of innovation and change Mechanisms for embedding change Evaluation and feedback processes Learning environment Organisational level: Organisational priorities Senior leadership and management support Culture Structure and systems History of innovation and change Absorptive capacity Learning networks External health system level: Policy drivers and priorities Incentives and mandates Regulatory frameworks Environmental (in)stability Inter-organisational networks and relationships

From Harvey et. al., 2016 [28].

**Table 2:** Focus group and interview details.

Focus Group /Interview	Institution Represented	Number of Participants	Disciplines/Backgrounds of Participants
Focus Group 1 (FG1)	Tertiary Care	7	Nursing, Gerontology
Focus Group 2 (FG2)	Primary/community care	9	Allied health (Occupational Therapy, Physiotherapy, Exercise Physiology)
Interview 1 (I1)	Primary/community care	3	Allied health, Nursing
Interview 2 (I2)	Primary/community care	2	Allied health (Occupational Therapy), Nursing
Interview 3 (I3)	Primary/community care	1	Allied health (occupational therapy)

**Table 3:** Themes and sub-themes generated, and associated i-PARIHS domains.

Theme	Sub-Theme	i-PARIHS Domain
*Legislation*	Policy/guidelines/legislation *	**Context** *External health system level* - Policy drivers and priorities, incentives and mandates, regulatory frameworks
*Staff-buy in*	Nothing new (leveraging and validating existing process)	**Innovation**—Compatibility (degree of fit within existing practice) **Innovation**—Relative advantage and compatibility
	Collaborative journey	**Recipients**—collaboration and teamwork **Context** *Local level*—learning environment *Organisational level*—learning networks
	Meaning and purpose	**Innovation**—clarity & usability
	Systematic approach to enhance practice & make it easier	**Innovation**—relative advantage
	Time & workloads as barriers	**Recipients**—time, resources and support
*Ancillary services and support*	Limited/restricted access to specialists for ‘the next step’	**Context**—External health system—environmental instability **Context**—external health system—interorganisational networks and relationships **Recipients**—existing networks
	Interorganisational roles and relationships	**Context**—external health system—inter organisational networks and relationships
*Tool availability*	Evidence base of standardised tested tools	**Innovation**—underlying knowledge sources
	Context appropriate	**Innovation**—compatibility and usability
	Validity of tool—relevance of the questions and the underpinning domains tapped into	**Innovation**—compatibility and usability
*Reliance on clinical experience*	Over-reliance/overconfidence potentially limiting	**Recipients**—values and beliefs
	Clinical experience to enhance/complement screening	**Recipients**—skills and knowledge
*Training and proficiency*	Training = experience = knowledge and confidence	**Recipients**—time resources and support, and skills and knowledge **Context** *Local level*—learning environment *Organisational level*—learning networks
	Lack of training or proficiency	**Context**—*Local level*—learning environment **Recipients**—skills and knowledge
*Patient experience as a motivator*	Observable & tangible benefits	**Recipient**—motivation
	Patient (dis)comfort & fear of negative outcomes	**Recipient**—motivation
*Spectrum of familiarity and implications for screening*		**Context**—External health system level

* Relevant to/identified from tertiary care setting.

**Table 4:** Summary of barriers and facilitators to cognitive screening as identified in two rural healthcare settings.

Barrier	Facilitator
Limited time Existing workloads and burden Subjective conclusions/assumptions based on over reliance on clinical knowledge/skills Limited availability of and access to specialists (for referral based on outcomes of screening) * Lack of confidence or screening related knowledge in workforce* Overfamiliarity with clients* Fear/stigma of outcome of screening *	Policy/legislation relating to screening Staff buy-in ○ Making existing process easier and validating practice. ○ Leverages off existing practice. ○ Creation of meaning and shared purpose associated with screening program. ○ Gradual change/implementation Collaborative implementation with staff where experiences and contributions valued Availability and awareness of context appropriate & valid screening tools Enhanced motivation through observing patient benefits Training undertaken/available to workforce ○ Enhanced individual knowledge and confidence Strong relationships and networks with & availability of external support organisations. ○ Knowledge of services available and contacts enhanced in rural setting * Clinical experience enhancing context/understanding of screening and results

* Enhanced in/unique to rural settings.

## Data Availability

The data presented in this study are not publicly available due to the potential for identification of research participants and in line with the conditions of ethics approval.

## Supplementary Materials

The following supporting information can be downloaded at: https://www.mdpi.com/article/10.3390/geriatrics7020035/s1, Interview Schedule.
