# Supplementary material for: Barriers and Facilitators to Screening for Cognitive Impairment in Australian Rural Health Services: A Pilot Study
Source: Geriatrics (Basel). 2022 Mar 22;7(2):35. doi: 10.3390/geriatrics7020035 (PMC9029066; doi:10.3390/geriatrics7020035)
Supplement: Supplementary file 1 [file geriatrics-07-00035-s001.zip › geriatrics-1619540-supplementary.pdf]

Overcoming barriers and harnessing facilitators to introduce, implement, and sustain effective Cognitive Impairment screening in regional Health Services

Focus Groups

Welcome

Thank you all for agreeing to be part of the focus group. We appreciate your willingness to participate.

Introduction

Purpose of Focus Group

We are here to discuss Cognitive Impairment (CI) screening of patients aged over 65. We would like to know about your experience with and perceptions of screening, including what is currently being done and what the barriers and facilitators to successful screening are. We aim to support successful implementation and sustainability of screening in rural and regional health services. Your feedback on the process and the experience will help us achieve this.

Ground Rules

1. We want **YOU** to do the talking.
    a. We would like everyone to participate and might call on you if we haven't heard from you in a while.
2. There are no right or wrong answers.
    a. Every person's experiences and opinions are important.
    b. Speak up whether you agree or disagree.
    c. We want to hear a wide range of opinions
3. What is said in this room stays here.
    a. We want people to feel comfortable sharing when sensitive issues come up.
4. We will be recording the group.
    a. We want to capture everything you have to say and ensure we can remember it correctly.
    b. The recording will be stored privately and only the researchers in this project will have access to it.
    c. We don't identify anyone by name in our report. You will remain anonymous.
5. Participation is voluntary. We will ask you to fill out a consent form before we start recording. If you want to stop participating at any point please let us know. Due to the nature of a focus group, we won't be able to delete anything said previously to that, but you won't have to participate any further.

Questions

There are a number of things that we want to cover today. As we only have 90 minutes, we might need to stop the discussion at some points to continue with the next question.

Part 1: Understanding of the external context

1. What is your understanding of the current National Safety and Quality Health Service (NSQHS) Standards and requirements around Cognitive Impairment screening of people aged over 65, and how this relates to your organisation?

2. Why do you think this is a requirement?

   (Note: we want to keep this question open to capture both the positive and negative perceptions around screening and CI)

Part 2: Current practice within the organisation

3. What is current practice around CI screening?
   Prompts:
   i. Which patients are being screened (selection criteria)?
   ii. Who conducts the screening (which staff)?
   iii. What tools/measures are in use?

4. What has determined this current practice?
   Prompts:
   i. History/precedence
   ii. The NSQHS standards (external environment)
   iii. Time
   iv. Knowledge/training/competency
   v. Personnel based (are they more personnel dependent in rural/regional services)
5. What has determined which measures are being used (if this is not covered in the preceding conversation)? And what are specific issues with the measures?
   Prompts:
   i. History/precedence
   ii. Time
   iii. Knowledge/training/competency

Part 3: Barriers to implementing screening

6. What are the key barriers to implementing cognitive screening of older (>65) patients (either thinking about what you have done in the past or compliance with the new standards)?
   Prompts:
   i. Staff attitudes/perceptions of costs and benefits
   ii. Time
   iii. Education/knowledge/competency
   iv. Workforce issues e.g. staff turnover
   v. Rural/regional issues

Part 4: Facilitators to implementing screening

7. What do you think might assist with implementing screening and/or increasing screening rates (i.e. possible facilitators)?
   Prompts:
   i. What has worked in the past and what hasn’t worked?
   ii. If you could see the benefits and outcomes of screening, would this be helpful?
   iii. What education and training would be required?

Other comments?

Thank you for your participation.
